# Patients’ experiences and expectations of chiropractic care: a national cross-sectional survey

**DOI:** 10.1186/s12998-014-0049-0

**Published:** 2015-01-16

**Authors:** Hugh MacPherson, Elizabeth Newbronner, Ruth Chamberlain, Ann Hopton

**Affiliations:** Department of Health Sciences, University of York, York, UK; Firefly Research & Evaluation and Visiting Fellow, Department of Health Sciences, University of York, York, UK; Firefly Research & Evaluation, North Yorkshire, UK

**Keywords:** Chiropractic, Patients’ expectations, Patients’ experiences, Risk, Benefit, Fitness to practice

## Abstract

**Background:**

Not enough is understood about patients’ views of chiropractic care. The aims of this research were to explore patients’ experiences and expectations, their perceptions of benefits and risks, and the implications for chiropractors’ continuing fitness to practise.

**Methods:**

Survey questions were formulated from existing literature, published guidance on good practice from the General Chiropractic Council, and from 28 telephone interviews and a small focus group with chiropractic patients using a semi-structured topic guide. In its final form, the survey elicited patients’ ratings on expectations regarding 33 aspects of care. In a national cross-sectional survey, a number of sampling methods were required as a consequence of the low practitioner response rate.

**Results:**

In total, 544 completed questionnaires were received from chiropractic patients, a lower response rate than expected (8%). The two main benefits that patients reported regarding their chiropractic care were reduced pain (92%) and increased mobility (80%). Of respondents, 20% reported unexpected or unpleasant reactions to their treatment, most commonly tiredness or fatigue (32%), and extra pain (36%). In most cases they expressed low levels of concern about these reactions. Patients’ expectations were met for most aspects of care. The four aspects of practice where expectations were least well met comprised: having more information on the cost of the treatment plan at the first consultation (80%); the chiropractor contacting the patient’s general practitioner if necessary (62%); having a discussion about a referral to another healthcare practitioner (62%); and providing a method for confidential feedback (66%).

**Conclusions:**

Overall, patients reported a high level of satisfaction with the benefits of their chiropractic care, although there is a likelihood of bias towards patients with a positive experience of chiropractic. There were no serious adverse reactions; however, patients reported concern about pain, tingling and numbness in the limbs after chiropractic. In general, patients’ expectations were being well met.

**Electronic supplementary material:**

The online version of this article (doi:10.1186/s12998-014-0049-0) contains supplementary material, which is available to authorized users.

## Background

There is a growing body of research into patients’ expectations and experiences (including adverse reactions) of complementary therapies. The OPEn Study [1] funded by the UK’s General Osteopathic Council explored patients’ expectations and experiences in relation to the benefits, risks and side effects of osteopathic treatment. It prioritised five areas of practice for the profession and the regulator to consider: the clinic environment; professionalism; treatment; relationship; and outcomes. It found a high level of satisfaction amongst patients consulting osteopaths working in private practice and most of the more widely held expectations were being met. Many of these related to the overall ‘customer experience’ but others were concerned with the therapeutic process and included the importance of “informing patients about what to expect in relation to treatment and outcomes including side effects” [1]. The study identified some gaps between expectations and delivery of care which could have a negative effect on the outcomes of care, and suggested that these gaps could be reduced by improving care and/or managing expectations better.

There has been relatively little work on the expectations of patients of chiropractic. One study conducted in Sweden [2] found that whilst patients and chiropractors had similar expectations in relation to key areas such as the chiropractor diagnosing and explaining the nature of the problems to the patients, there were other important areas where expectations differed. In particular, patients were more likely than their chiropractor to expect a rapid (i.e. within one to two treatments) improvement in their condition. They were also more likely to expect to be given advice about how to manage their problem and exercises to do between treatments. Interestingly, the OPEn Study highlighted this as an area where the level of unmet positive expectation was relatively high.

In a Dutch study [3] on the benefits and risks of chiropractic care for neck pain, 529 patients provided data that showed that adverse reactions are rarely severe in nature and for most patients the benefits outweighed the risks. Two studies [4] commissioned by the UK’s General Chiropractic Council (GCC) in 2009/10 brought together information from clinical research about the risks of chiropractic, and examined the possible costs of adverse reactions and sub-optimal outcomes. They concluded that ‘suboptimal outcomes’, such as delayed or missed diagnosis, missed recognition of contra-indications, inadequate care-management and poor record keeping, were of more concern (and had greater cost implications) than significant adverse reactions.

Whilst much can be learned from these and other studies, both from their direct work with patients and the wider literature reviews, the GCC identified an evidence gap in relation to UK chiropractic patients’ views and expectations. Moreover the GCC was also reviewing its approach to the revalidation of registrants, and as part of this process wanted to gain a fuller understanding of patients’ views of chiropractic care, in particular people’s assessment of the risks and benefits of chiropractic treatment, their expectations of chiropractors, and their experience of them. In this context, the GCC commissioned independent researchers from Firefly Research & Evaluation, in partnership with the Department of Health Sciences at the University of York, to carry-out research with a focus on the experiences and expectations of patients and the extent to which these expectations were met. The results of the research outlined in this report were designed to inform the GCC’s work on revalidation. This paper presents an overview of the survey results.

## Methods

### Design and setting of the qualitative interviews and focus group

We used qualitative methods as a preparation for designing and conducting a national survey. Three areas (Cardiff, the Scottish Borders, and Mansfield/Chesterfield) were chosen for the interviews and focus group to reflect city, town and rural communities. The chiropractors in each area were asked to assist with the study by inviting up to 10 of their current and former patients (including as far as possible a mix of age, gender, ethnicity and condition or disability) to take part in either a focus group, a telephone interview or face-to-face interview. In total, 12 chiropractors agreed to be involved and through them 30 patients gave written consent to take part in the study: 56% of these patients were female and 73% were aged under 65. Twenty-eight patients chose to take part in individual telephone interviews and two attended a focus group (in Mansfield/Chesterfield). The interviews and focus group were conducted by two experienced female health services researchers (LN, RC), using a semi-structured topic guide covering four main topics: ‘choosing your chiropractor’; ‘expectations’; ‘risks and benefits’; and ‘fitness to practice’. The topic guide used in the interviews provided an initial framework which was then expanded. The interviews were digitally recorded and typically lasted around 40 minutes. The interviews and focus group recordings were transcribed in full and the data were then analysed thematically [5] by two researchers (LN, RC). Ethical approval was obtained from the Research Governance Committee of the University of York’s Department of Health Sciences (Ref no. HSRGC/2012/06)

### Development of the questionnaire

The questionnaire used in the national cross-sectional survey was developed from reviewing the existing literature about patients’ experiences and expectations of manual therapies, the General Chiropractic Code of Practice and Standard of Proficiency [6] and the telephone interviews and the focus group, as described above. The sections of the questionnaire were informed by five questions that were set out by the GCC to inform the research:

- What do patients of chiropractors see as the benefits of receiving chiropractic care?
- What do patients see as the potential risks of receiving chiropractic care?
- Has their perception of benefits and risks changed over time? If so, how?
- What has influenced their perceptions of the benefits and risks?
- Once a chiropractor is on the GCC register, what do patients expect will happen to assure an Individual chiropractor’s continuing fitness to practise?

The core of the questionnaire focused on patients’ experiences and expectations of chiropractic care and treatment at different stages in their contact with the chiropractor. We also asked patients directly about benefits, adverse reactions and what systems would reassure them that their chiropractor was keeping their knowledge and skills up to date. We tested the first draft of the questionnaire with a small number of patients who had taken part in the interviews and volunteered to assist us at this stage. Building on their comments and following discussions with colleagues at the GCC and within the research team, we developed a second draft of the questionnaire with was then tested with a sample of patients from a single chiropractic practice. Following this second pilot, the final version of the questionnaire was developed and agreed with colleagues at the GCC. A copy of the final questionnaire is attached in Additional file 1.

### Survey participants

During November 2012 a random sample of 600 chiropractors (drawn from the GCC’s database of registrants; n =960) were invited to participate in a national cross-sectional survey by distributing a questionnaire (available in paper-based form or electronically) to at least 10 of their current and former patients: four to current patients who were receiving regular chiropractic care over the past three months or more; two to new patients (i.e. at their first consultation); two to former patients who ceased treatment within the past 6 months by mutual agreement with the chiropractor and/or because the presenting problem was resolved; two to former patients who ceased treatment within the past 6 months but who ended their treatment unilaterally (i.e. before, in the chiropractor’s view, the presenting problem was resolved). Practitioners were also asked to try to include a mix of age, gender, ethnicity and disability. In the first approach involving 600 practitioners, 47 chiropractors agreed to take part (8%). We subsequently contacted an additional sample of 360 chiropractors and of these 21 agreed to take part (6%). A further two chiropractors volunteered to be involved when the British Chiropractic Association (BCA) sent an email to its members. This resulted in a total of 70 chiropractors who agreed to help recruit patients for the survey. Information about the survey was also circulated by email to chiropractic patients registered with Care Response, an organisation that supports the collection of routine patient-related data (http://www.careresponse.co.uk/)

### Data analysis

Data was entered onto an Excel spreadsheet and two researchers (Martin Baxter, Liz Newbronner) conducted a random audit of 10% of questionnaires to check for accuracy. Analysis of the survey data was conducted to characterise the survey respondents to assess the distribution of age within the sample, and the analysis included calculation of the mean, median and interquartile range values and the calculation of Z scores to assess the distribution of ages. Demographic data, comprising sex, ethnicity, educational attainment, living environment and nationality, were analysed using frequencies and proportions. Other survey data regarding the experiences and expectations of chiropractic treatment were also analysed using frequencies and proportions. Associations between adverse reactions were analysed using Chi-squared statistics. Questions that were not answered (i.e. missing data) were also reported as frequencies and proportions.

## Results

### Patient participation rates and patient profile

We sent 1075 information sheets and questionnaires to the 70 recruited chiropractors and asked them to distribute these to their patients. This generated 401 usable questionnaires, a 37% response rate. A total of 5167 patients registered with 36 Care Response member chiropractors were emailed and 112 (2%) patients completed the survey through this route. In addition, the Chiropractic Patients Association also informed its members of the survey (N = 350) and 27 (9%) members and four of their friends and family responded as a result of this. Together all these recruitment methods generated a total of 544 respondents. Excluding the four family and friends, for whom we have no denominator, this represents an overall response rate of 8% (540/6592). The majority of questions (82%) had been answered; however, five questions (questions 40–44) contained between 16% and 20% of missing data. These questions with the greater proportion of missing data corresponded with the less well met expectations.

Of the 544 respondents, 360 (66%) were women; 4 (0.7%) did not give their gender (Table 1). The age distribution showed a significant negative skew (z = −2.12). The median age of respondents was 54.5 years (interquartile range = 43–71); yet when examined more closely, the age profile for women followed a normal distribution whilst the age profile for men showed a significant negative skew (z = −2.810) whereby 70% of the male participants were aged 50 and over. The majority of respondents lived in England (89%), classified their ethnic origin as white (96%), and lived within a city or town environment (58%). With regard to their highest level of educational qualification, 50% of the respondents had either first degrees or second degrees. This compares with 30% of the UK working age population having a first degree or higher [7].

**Table 1:** **Demographic of chiropractic patients surveyed (Total n = 544)**

**Questions asked**		
**What is your age? (n = 544)**	**Years**	
Range =	16-87	
Median=	54.50	
Interquartile range 1; Interquartile range 3 =	43 ; 71	
Missing	0	
**Are you? (n = 540)**	**n =**	**(%)**
Male	180	33%
Female	360	66%
Missing	4	0.7%
**What is your ethnicity? (n = 543)**		
White	519	96%
Black or Black British	6	1%
Asian or Asian British	6	1%
Chinese or Chinese British	2	0.4%
Mixed heritage	0	0
Other ethnic group	8	1.4%
Missing	1	0.2%
**Do you consider yourself to have a disability? (n =536)**		
Yes	44	8%
No	492	91%
Missing	8	1.4%
**What is your highest level of academic education/attainment? (n = 527)**		
No academic qualifications	38	7%
GCSE or equivalent (e.g. O level, CSE, NVQ1)	96	18%
A levels or equivalent (e.g. NVQ2-3, BTec certificate, City and Guilds crafts)	117	22%
BA or BSC degree or equivalent (e.g. NVQ4, BTEC diploma, City and Guilds level 3+, nursing or teaching qualifications)	193	36%
Master’s Degree, PhD, Post graduate certificate or NVQ level 5	79	15%
Other	4	0.7%
Missing	18	3%
**How would you describe the area that you live in? (n = 534)**		
City/Urban area	50	9%
Town or suburb	267	49%
Village or rural area	217	41%
Missing	10	1.8%
**Which of the four UK nations do you live in? (n = 535)**		
England	484	90%
Scotland	13	2%
Wales	28	5%
Northern Ireland	8	1.4%
Missing	9	1.6%

### Experiences of chiropractic care

Before receiving chiropractic treatment, 58% of patients had limited knowledge of what the treatment involved, 41% were unsure of the likely benefits and 71% had little knowledge of the possible reactions to treatment (Table 2). Over half (53%) attended every other month, 25% were no longer receiving treatment and 59% no longer needed treatment. One third of the patients had changed their chiropractor at some point in the past with the main reasons being: linked to the patient moving to a new area; finding a chiropractor at a more convenient location; or the chiropractor moving away. Eight people (5%) said that the approach or manner of the chiropractor did not suit them or the treatment was not benefiting them.

**Table 2:** **Experiences of chiropractic treatment (n = 544)**

**Statements**		
**Before you first had chiropractic treatment, how much did you know about:**		
**a. What the treatment involved**	n =	%
I knew very little	318	58%
Some knowledge	148	27%
I knew a lot	75	14%
Missing	2	0.4%
**b. The likely benefits of the treatment**		
I knew very little	224	41%
Some knowledge	182	33%
I knew a lot	134	25%
Missing	2	0.4%
**c. Possible reactions to the treatment**		
I knew very little	387	71%
Some knowledge	112	21%
I knew a lot	41	7%
Missing	2	0.4%
**Approximately how many chiropractic treatments have you had in the last 12 months?**	n = treatments	
Range =	0-55	
Median =	6	
Interquartile range 1; Interquartile range 3 =	4 ; 10	
**Are you currently receiving treatment?**	n =	%
YES	409	75%
NO	135	25%
**Reasons given in answer to the question, “If ‘NO’, why did you stop chiropractic treatment?” (n = 135)**		
	n =	%
The problem being treated improved/got better, I no longer needed treatment	79	59%
The problem being treated has improved and I am currently able to manage it myself	47	35%
I did not feel the treatment was benefitting me	6	4%
I was unhappy with the chiropractor’s approach/manner	2	1.5%
I had an unpleasant reaction to treatment	0	0%
I was unhappy with the cost of treatment	3	2%
I am currently unable to afford treatment	7	5%
Other health problems have prevented me from having chiropractic treatment	1	0.7%
NB. 10 people (7%) gave more than one reason for stopping chiropractic treatment	145	107%

### Experiences of benefits and adverse reactions

The majority of patients surveyed reported benefits associated with chiropractic. These benefits included a reduction in the pain (92%) and an improvement in mobility (80%) (Table 3). More than half reported that chiropractic treatment had an ongoing effect of reducing future problems (55%) and provided a better understanding of their health problem (53%).

**Table 3:** **Experiences of chiropractic treatment: main benefits of treatment (n = 544)**

	**n =**	**%**
It has reduced or removed the pain I was experiencing.	503	92%
It has increased my mobility/flexibility.	434	80%
It has helped me maintain my general health and wellbeing.	344	63%
It is helping to prevent or reduce future problems.	297	55%
It has given me a better understanding of my health problem.	286	53%
It has increased my ability/confidence to manage my health problem.	239	44%
It has enabled me to return to work, sport or other activities.	204	38%

Approximately 20% (n = 110) of respondents reported one or more reactions to treatment that they found unexpected or unpleasant. Of a total of 153 reactions, among respondents 45% (n = 49) had one reaction, 34% (n = 37) had two reactions and the remainder had 3 or more (n = 15) (Table 4). Where patients reported on more than one type of reaction, they reported a single level of concern associated with their reactions. Extra pain and/or radiating pain was the symptom associated with the greatest level of concern (n = 19). Where patients had more than one reaction, extra pain was more commonly combined with tingling and stiffness.

**Table 4:** **Experiences of chiropractic treatment: frequency of adverse reactions and reported level of concern (n = 110 of 544 patients)**

**Reaction**	**Level of concern (n =)**		
	**Of lower concern (1,2)**	**Of greater concern (3,4,5)**	**Total number of reactions**
Tiredness or fatigue	33	2	35
Headache	19	4	23
Extra Pain and/or Radiating Pain	21	19	40
Tingling/numbness in legs or arms	3	9	12
Stiffness	13	7	20
Dizziness or light headedness	13	6	19
Nausea	1	3	4
Total	103	50	153

NB. Where patients reported on more than one type of reaction, they reported a single level of concern associated with their reactions.

### Expectations of chiropractic care

Patients reported their expectations prior to the first consultation and what they experienced in practice (Table 5). Where patients responded ‘Yes’ to the question ‘Did it happen’ their expectation was met, but a response of ‘No’ signified their expectation was not met. For these questions, more than 80% of patients expected these aspects of care to happen and they did happen, which suggests there was a high level of satisfaction with this stage of the ‘patient journey’.

**Table 5:** **Expectations of chiropractic care: responses to questions related to ‘before seeing the chiropractor’ (n = 544); n/a = not answered**

**Statements**	**Expected**			**Did it happen?**		
	**Yes**	**No**	**n/a**	**Yes**	**No**	**n/a**
Q11. Before seeing the Chiropractor I expect to be given general information about chiropractic treatment and what it involves.	91%	8%	1%	92%	7%	1%
Q12. Before seeing the Chiropractor I expect to be given general information about possible reactions (both positive and negative) to chiropractic treatment.	82%	15%	3%	82%	15%	3%
Q13. Before seeing the Chiropractor I expect to be given information about what will happen at my first consultation.	91%	7%	2%	91%	7%	2%
Q14. Before seeing the Chiropractor I expect to be told about the cost of treatments.	97%	2%	1%	95%	4%	1%
Q15. Before seeing the Chiropractor I expect to be told how long the first consultation is likely to last.	90%	8%	1%	91%	7%	2%
Q16. Before seeing the Chiropractor I expect to fill-in and sign a consent form for the first consultation.	83%	13%	3%	90%	6%	4%
Q17. Before seeing the Chiropractor I expect to provide my General Practitioner's name and contact details.	83%	15%	2%	91%	6%	3%

Similar results were obtained for the patients’ first consultation (Table 6). An area where there was a noticeable gap between expectations (86%) and experience (80%) was in regard to an explanation of the cost of the treatment plan at the first consultation. Though there was a small difference of 3% between the expectation the chiropractor would talk about the possibility of adverse reactions and what happened, 13% of patients reported that this did not happen.

**Table 6:** **Expectations of chiropractic care: responses to questions about the first consultation (n = 544); n/a = not answered**

**Statements**	**Expected**			**Did it happen?**		
	**Yes**	**No**	**n/a**	**Yes**	**No**	**n/a**
Q18. At the First Consultation I expect to be given time to tell the chiropractor about my problem and how it was affecting me.	99%	0%	1%	99%	0%	1%
Q19. At the First Consultation I expect the chiropractor to take a detailed account of my personal case history.	96%	3%	1%	96%	1%	2%
Q20. At the First Consultation I expect to be given a gown and/or towels to cover up if I had to undress.	83%	12%	4%	84%	9%	7%
Q21. At the First Consultation I expect to be able to undress and dress in privacy.	90%	6%	5%	88%	5%	7%
Q22. At the First Consultation I expect the chiropractor to explain why any further investigations (e.g. X-Rays) were necessary and any risks associated with them.	72%	18%	10%	67%	20%	14%
Q23. At the First Consultation I expect the chiropractor to give me a diagnosis or rationale for my care.	93%	5%	2%	95%	3%	3%
Q24. At the First Consultation I expect the chiropractor to explain what treatment I will need (e.g. the type, frequency and duration of treatment).	97%	2%	1%	94%	4%	2%
Q25. At the First Consultation I expect the chiropractor to talk to me about any possible adverse reactions to the treatment.	87%	9%	3%	83%	13%	3%
Q26. At the First Consultation I expect the chiropractor to talk to me about the likely success of the treatment.	93%	6%	2%	91%	7%	2%
Q27. At the First Consultation I expect the chiropractor to explain what the cost of the agreed treatment plan would be.	86%	12%	3%	80%	17%	3%
Q28. At the First Consultation I expect the chiropractor to give me time to ask questions about the proposed treatment plan.	96%	3%	1%	94%	4%	2%

Questions were asked of respondents about on-going treatment processes (Table 7). In most cases, more than 90% of patients’ expectations and experiences corresponded well. In some areas, chiropractors exceeded their patients’ expectations, for example by displaying information about their length of time in practice and about their special interests or additional skills. In the survey, 99% of the respondents expected that their chiropractor would allow sufficient time for their consultation and this expectation was largely met, with 97% saying that it had happened. People responding to the survey had slightly lower expectations (85%) relating to when treatment should be reviewed.

**Table 7:** **Expectations of chiropractic care: responses to questions related to on-going treatment (n = 544); n/a = not answered**

**Statements**	**Expected**			**Did it happen?**		
	**Yes**	**No**	**n/a**	**Yes**	**No**	**n/a**
Q29. I expect to be given time to tell the chiropractor about how I felt after my last treatment, and discuss any problems or concerns.	98%	1%	0%	97%	1%	1%
Q30. I expect the chiropractor to ask me if there has been any change in my condition, general health or medication.	97%	2%	1%	97%	2%	1%
Q31. I expect the chiropractor to allow sufficient time for the consultation.	99%	0%	1%	97%	2%	1%
Q32. I expect the chiropractor to talk to me about further treatment options.	91%	6%	2%	87%	9%	4%
Q33. I expect the chiropractor to allow me time to decide what I wish to do about future treatment.	92%	5%	3%	91%	5%	4%
Q34. I expect the chiropractor to agree with me when my treatment should be reviewed.	85%	11%	4%	84%	10%	6%
Q35. I expect the chiropractor to give me advice about how I manage my problems/symptoms between treatments.	97%	2%	1%	96	3%	1%

When considering referral to other agencies, 86% of survey respondents expected that the chiropractor would refer them to other healthcare practitioners if appropriate and 87% expected them to contact their general practitioner, should this be needed (Table 8). In both cases, nearly two-thirds (62%) said this happened although 20% and 18% respectively did not answer the questions (q40, q41). It is not clear whether this relates to the expectation not being delivered or whether a referral was not required. Another area where there appeared to be a gap between expectations (76%) and experiences (66%) was with regard to a system for patients to provide confidential feedback; between 16% and 20% of patients did not answer these questions. A high percentage (97%) of survey respondents expected the chiropractor to provide advice on how to manage symptoms between treatments and this expectation was largely met, with 96% of respondents saying they had been given this type of support.

**Table 8:** **Expectations of chiropractic care: assuring chiropractors are ‘Fit to practice’ responses to questions related to the chiropractor’s knowledge and experience; (n=544); n/a = not answered**

**Statements**	**Expected**			**Did it happen?**		
	**Yes**	**No**	**n/a**	**Yes**	**No**	**n/a**
Q36. I expect that information about the chiropractor's qualifications and registration will be displayed in the clinic/available in leaflets/included on the practice website.	90%	8%	2%	95%	4%	2%
Q37. I expect that information about the chiropractor's experience (e.g. length of time in practice) will be displayed in the clinic/available in leaflets/included on the practice website.	76%	22%	3%	82%	13%	6%
Q38. I expect that information about the chiropractor's special interests or additional skills (e.g. soft tissue massage) will be displayed in the clinic/available in leaflets/included on the practice website.	69%	26%	5%	75%	17%	9%
Q39. I expect that information about how the chiropractor is maintaining/improving their professional knowledge (e.g. further training) will be displayed in the clinic/included on the practice website.	56%	38%	6%	59%	28%	13%
Q40. I expect that if my problem is not improving with chiropractic treatment and/or I have other health needs, the chiropractor will discuss referral to another healthcare practitioner.	86%	8%	6%	62%	18%	20%
Q41. I expect that, if necessary, and with my consent, the chiropractor will contact my GP.	87%	10%	4%	62%	20%	18%
**Responses to statements related to patient feedback and complaints**						
Q42. I expect the chiropractic practice to have a clear system to enable me to provide confidential feedback (whether positive or negative).	76%	20%	4%	66%	18%	16%
Q43. I expect to be given information about the practice's complaints procedure.	54%	39%	7%	44%	38%	18%
Q44. I expect to be told about my right to refer a complaint to the General Chiropractic Council and be given the GCC's contact details.	51%	41%	9%	40%	40%	20%

### Expectations with regard to fitness to practice

With regards to the chiropractors’ fitness to practice, almost half of the patients responding to the survey (49%) would feel reassured if patients were provided with information about the chiropractors’ on-going training and development, whereas 19% disagreed and 30% were undecided. Between half and two-thirds of patients (61%) agreed that chiropractors should review their practice regularly (61%), be assessed by an independent assessor (66%) and have a practice-level system for gathering and showing patient feedback (59%). Half (50%) agreed that there should be a national system for gathering patient feedback and that patients could access.

## Discussion

In a survey comprising 544 patients, the positive experiences reported were the benefits of reduced pain and increased mobility and an understanding of how to maintain the improvements gained. There were no serious adverse reactions. Regarding minor reactions to treatment, some people reported greater concern about extra pain, tingling and numbness, whereas tiredness, fatigue and headache were of low concern. The survey showed that the patients’ expectations of advice on how to manage symptoms between treatments were largely met. There were four areas where patients had relatively high expectations (i.e. >75% expected an aspect of care to happen) but these were not met for a significant proportion of patients (between 10 to 25%). These were: if necessary and with consent, the chiropractor would contact the patient’s general practitioner; if the problem is not improving or the patient has other health needs the chiropractor will discuss referral to another healthcare practitioner; having the cost of the treatment plan explained at the first consultation; and the chiropractic practice having a system for patients to provide confidential feedback.

### Strengths and limitations of the study

In terms of strengths, the study was conducted by a research group that was independent of associations or regulatory bodies associated with chiropractic. This independence meant that the study was not biased by prior agendas or partiality towards particular styles of chiropractic. The use of interviews with patients receiving chiropractic care prior to the national survey helped shape a more appropriate and targeted questionnaire. The piloting of the survey questions helped clarify the focus of the survey and reduced ambiguity. Nevertheless, the survey did require patients to recall their first consultation and so there is a risk of recall bias.

For many questions the level of expectation is high and the estimation of expectations being met may be reflected accurately. However, where the level of expectation is below 80% there may be a possibility of a substantial over-estimation of expectations being met. Furthermore, the missing data on questions relating to referral and the patients’ complaint procedure may also reflect that the question was not applicable to them, because they had not previously considered the questions and therefore found it difficult to answer whether or not they expected to be told of either referral or complaints procedures.

A limitation of the survey was related to the difficulty we had in engaging chiropractors in the study, though we did eventually recruit 70, two of whom were not contacted directly. We only achieved a response rate of 7% (n = 68) from the 960 chiropractors that we contacted directly. Moreover the response rate from their patients was also poor, with 37% of the questionnaires sent to chiropractors returned in paper form or on-line by their patients. It is possible that, despite the guidance provided by the research team, the chiropractors could have selected patients who they felt would provide a positive response, thereby biasing the sample. Therefore it could be argued that among respondents, largely consisting of patients selected by their own chiropractor or those who have joined a Chiropractic Patient’s Association, attitudes towards chiropractic were biased towards being more favourable.

There is some research evidence [8] that the overall population of chiropractic patients is fairly evenly split between women and men. We also noted the relatively high proportion of participants with either first degrees or second degrees compared to the UK working age population and speculate that this may reflect an affordability issue, in that chiropractic is more likely to be used by professional classes. In terms of how representative the patients involved in this study are of all patients who seek chiropractic care, it is important to sound a note of caution. The nature of this study means that most of the patients who contributed were either currently receiving treatment or had had on-going chiropractic care in the recent past. Those patients who ceased chiropractic care after a small number of treatments, perhaps because they were unhappy with their care may well be under-represented. Nevertheless, our study data also suggests that where patients are unhappy with their treatment, this does not necessarily deter them from seeking further treatment from another chiropractor.

### Comparison to previous literature

The positive experiences related to expectations of chiropractic care reported in this study are consistent with a previous quantitative study of expectations related to osteopathic treatment,[9] in which the majority of people were satisfied with the treatment they received and their expectations of care were largely met. Also consistent with qualitative evidence within the osteopathic literature is that the most important expectations relate to the themes of individual agency, professional expertise, customer experience, therapeutic process and interpersonal relationships [10]. In a survey of chiropractic patients in Sweden, patients were found to have lower expectations of the chiropractic treatment than the chiropractors had, but higher expectations of being given advice and exercises than the chiropractors had [2]. Moreover, patients expected to get better faster than the chiropractors expected them to. We found that patients’ expectations about being given advice about managing symptoms were largely met. In another study involving chiropractic patients, the researcher found that patients had an inaccurate or rudimentary understanding of the mechanisms that underpin chiropractic treatment, were not particularly interested in how it worked and despite the explanations of the chiropractor, patients showed only a limited understanding of the mechanisms [11]. In our study more than 90% expected to be given information about chiropractic and what it involved and to be given a diagnosis and rationale for their treatment. However, our survey did not ask whether the patients understood any of the information provided. There remains a gap in the literature, as previously identified [2], on whether unmet expectations contribute to lower levels of effectiveness.

### Implications for practice

With regard to clinical practice, the less well met expectations can provide guidance on how chiropractic care could be improved from the perspective of patients. These areas where there could be some improvement include: allowing sufficient time for the consultation; being told about the cost of treatments; having the treatment needed explained; being given time to ask questions about the treatment plan; being told about the likely success of the treatment and adverse reactions; being told about further treatment options; and being able to undress and dress in privacy. A more substantial expectation/experience gap that could be addressed by changes in practice include: if necessary and with consent, the chiropractor would contact the patient’s general practitioner; if the problem is not improving or the patient has other health needs the chiropractor will discuss referral to another healthcare practitioner; having the cost of the treatment plan explained at the first consultation; and the chiropractic practice having a system for patients to provide confidential feedback. These gaps between expectations and delivery of care are mostly a consequence of poor communication and may lead to negative effects on outcomes of care and/or patient dissatisfaction. By closing the gaps, chiropractors individually and as a profession can improve their clinical practice. In terms of future research, this paper provides a platform from which a more comprehensive survey can be conducted, with better methods of recruiting chiropractic patients to improve the response rate and to limit selection bias.

## Conclusion

In general, patients reported a high level of satisfaction with the benefits of their chiropractic care, most commonly related to reduced pain and increased mobility. There were no serious adverse reactions. Among the minor reactions reported, there was greater concern about extra pain, tingling and numbness attributed to chiropractic and a lower level of concern about reactions of tiredness or fatigue. As reported by patients, expectations were largely met for most of the 33 aspects of practice. The areas where expectations were less well met were mostly due to inadequate or limited communication. By addressing these areas, chiropractors can improve the quality of their clinical practice and delivery of patient care.

## Additional files

Additional file 1:
**Questionnaire for national survey -final.pdf, 339 K** **http://www.chiromt.com/imedia/1336971084126552/supp1.pdf** **.**
