# Supplementary material for: Patients’ experiences and expectations of chiropractic care: a national cross-sectional survey
Source: Chiropr Man Therap. 2015 Jan 16;23:3. doi: 10.1186/s12998-014-0049-0 (PMC4307916; doi:10.1186/s12998-014-0049-0)
Supplement: Additional file 1: — Questionnaire for national survey -final.pdf, 339 K http://www.chiromt.com/imedia/1336971084126552/supp1.pdf . [file 12998_2014_49_MOESM1_ESM.pdf]

Firefly ILLUMINATING RESEARCH

General Chiropractic Council

THE UNIVERSITY of York
Department of Health Sciences

National Survey of Chiropractic Patients

Section A: Your Experience of Chiropractic Treatment

1.	Before you first had chiropractic treatment, how much did you know about: (please circle)	I knew very little				I knew a lot
a.	What the treatment involved?	1	2	3	4	5
b.	The likely benefits of the treatment?	1	2	3	4	5
c.	Possible reactions to the treatment?	1	2	3	4	5

2.	Approximately how many chiropractic treatments have you had in the last 12 months?		
3.	Are you currently receiving chiropractic treatment? (please circle)	Yes	No

4.	If No, why did you stop chiropractic treatment? (tick all that apply)	
	The problem being treated improved/got better and I no longer need treatment	
	The problem being treated has improved and I am currently able to manage it myself	
	I did not feel the treatment was benefiting me	
	I was unhappy with the chiropractor's approach/manner	
	I had an unpleasant reaction to treatment	
	I was unhappy with the cost of treatment	
	I am currently unable to afford treatment	
	Other health problems have prevented me having chiropractic treatment	
Other (please describe)		

5.	For you, what have been the main benefits of chiropractic treatment? (tick all that apply)	
	It has reduced or removed the pain I was experiencing	
	It has increased my mobility/flexibility	
	It has enabled me to return to work, sport or other activities	
	It has helped me maintain my general health and wellbeing	
	It is helping to prevent or reduce future problems	
	It has given me a better understanding of my health problem	
	It has increased my ability/confidence to manage my health problem	
Other (please describe)		

6.	Have you ever had an unexpected or unpleasant reaction to chiropractic treatment? (please circle)	Yes	No

7. If Yes, which reaction/s have you experienced? (tick all that apply)			
Tiredness or fatigue		Stiffness	
Headache		Dizziness or light headedness	
Extra Pain and/or Radiating Pain		Nausea	
Tingling/numbness in legs or arms		Other (please describe)	

	Not concerned				Very concerned
8. How concerned were you by this reaction? (please circle)	1	2	3	4	5

9.	Have you ever changed chiropractor? (please circle)	Yes	No
10.	If Yes, what was the reason? (please describe)		

Section B: Your experience of chiropractic care and treatment
For each question please circle Yes or No in **BOTH** the 'Expected?' and 'Did it happen?' columns

	**Before Seeing the Chiropractor**	Expected?		Did it happen?	
11.	I expect to be given general information about chiropractic treatment and what it involves	Yes	No	Yes	No
12.	I expect to be given general information about possible reactions (both positive and negative) to chiropractic treatment	Yes	No	Yes	No
13.	I expect to be given information about what will happen at my first consultation	Yes	No	Yes	No
14.	I expect to be told about the cost of treatments	Yes	No	Yes	No
15.	I expect to be told how long the first consultation was likely to last	Yes	No	Yes	No
16.	I expect to fill-in and sign a consent form for the first consultation	Yes	No	Yes	No
17.	I expect to provide my General Practitioner's name and contact details	Yes	No	Yes	No
	**At the First Consultation**	Expected?		Did it happen?	
18.	I expect to be given time to tell the chiropractor about my problem and how it was affecting me	Yes	No	Yes	No
19.	I expect the chiropractor to take a detailed account of my personal case history	Yes	No	Yes	No
20.	I expect to be given a gown and/or towels to cover up if I had to undress	Yes	No	Yes	No
21.	I expect to be able to undress and dress in privacy	Yes	No	Yes	No
22.	I expect the chiropractor to explain why any further investigations (e.g. X-Rays) were necessary and any risks associated with them	Yes	No	Yes	No
23.	I expect the chiropractor to give me a diagnosis or rationale for my care	Yes	No	Yes	No
24.	I expect the chiropractor to explain what treatment I will need (e.g. the type, frequency and duration of treatment)	Yes	No	Yes	No
25.	I expect the chiropractor to talk to me about any possible adverse reactions to the treatment	Yes	No	Yes	No
26.	I expect the chiropractor to talk to me about the likely success of the treatment	Yes	No	Yes	No
27.	I expect the chiropractor to explain what the cost of the agreed treatment plan would be	Yes	No	Yes	No
28.	I expect the chiropractor to give me time to ask questions about the proposed treatment plan	Yes	No	Yes	No

	On-Going Treatment	Expected?		Did it happen?	
29.	I expect to be given time to tell the chiropractor about how I felt after my last treatment, and discuss any problems or concerns	Yes	No	Yes	No
30.	I expect the chiropractor to ask me if there has been any change in my condition, general health or medication	Yes	No	Yes	No
31.	I expect the chiropractor to allow sufficient time for the consultation	Yes	No	Yes	No
32.	I expect the chiropractor to talk to me about further treatment options	Yes	No	Yes	No
33.	I expect the chiropractor to allow me time to decide what I wish to do about future treatment	Yes	No	Yes	No
34.	I expect the chiropractor to agree with me when my treatment should be reviewed	Yes	No	Yes	No
35.	I expect the chiropractor to give me advice about how I manage my problem/symptoms between treatments	Yes	No	Yes	No
	**Chiropractor's Knowledge and Experience**	Expected?		Did it happen?	
36.	I expect that information about the chiropractor's qualifications and registration will be displayed in the clinic/available in leaflets/included on the practice website	Yes	No	Yes	No
37.	I expect that information about the chiropractor's experience (e.g. length of time in practice) will be displayed in the clinic/available in leaflets/included on the practices website	Yes	No	Yes	No
38.	I expect that information about the chiropractor's special interests or additional skills (e.g. soft tissue massage) will be displayed in the clinic/available in leaflets/included on the practice website	Yes	No	Yes	No
39.	I expect that information about how the chiropractor is maintaining/ improving their professional knowledge (e.g. further training) will be displayed in the clinic/included on the practice website	Yes	No	Yes	No
40.	I expect that if my problem is not improving with chiropractic treatment and/or I have other health needs, the chiropractor will discuss referral to another healthcare practitioner	Yes	No	Yes	No
41.	I expect that, if necessary, and with my consent, the chiropractor will contact my GP	Yes	No	Yes	No
	**Patient Feedback and Complaints**	Expected?		Did it happen?	
42.	I expect the chiropractic practice to have a clear system to enable me to provide confidential feedback (whether positive or negative)	Yes	No	Yes	No
43.	I expect to be given information about the practice's complaints procedure	Yes	No	Yes	No
44.	I expect to be told about my right to refer a complaint to the General Chiropractic Council and be given the GCC's contact details	Yes	No	Yes	No

Further comments

Section C: Assuring Chiropractors are 'Fit to Practise'

We are interested in what would reassure you that your chiropractor is keeping their knowledge and skills up to date. Please indicate your level of agreement with the following statements		Strongly disagree				Strongly agree
45.	The chiropractor should provide patients with up to date information about their on-going training and development	1	2	3	4	5
46.	The chiropractor should review their practise regularly (e.g. every 3 years) with support from another experienced chiropractor	1	2	3	4	5
47.	The chiropractor should be reassessed regularly (e.g. every 3 years) by an independent assessor to ensure they are keeping up to date	1	2	3	4	5
48.	The chiropractor should have a system for gathering patient feedback and patients should be able to see the feedback recorded	1	2	3	4	5
49.	There should be a national system for gathering patient feedback and patients should be able to see the feedback recorded	1	2	3	4	5
Further comments:						

Section D: About You

50.	Are you?	Male	Female
51.	What is your age?		

52. What is your ethnicity? (please circle or tick one)		
White	Black or Black British	Asian or Asian British
Chinese or Chinese British	Mixed heritage	other ethnic group

53.	Do you consider yourself to have a disability?	Yes	No

54. What is your highest level of academic education/attainment? (please tick)			
No academic qualifications		GCSE or equivalent (e.g. O Level, CSE, NVQ1)	
A levels or equivalent (e.g. NVQ 2-3, BTec certificate, City and Guilds crafts)		BA or BSc degree or equivalent (e.g. NVQ 4, BTEC diploma, City and Guilds level 3+, nursing or teaching qualification)	
Masters Degree, PhD, Post graduate certificate or NVQ 5		Other (please give details)	

55. How would you describe the area you live in? (please circle or tick one)		
City/Urban area	Town or suburb	Village/Rural area

56. Which of the four UK nations do you live in? (please circle or tick one)			
England	Scotland	Wales	Northern Ireland

Many thanks. Please return in the freepost envelope provided
